# Ambient Air Pollution and Low Birth Weight in Connecticut and Massachusetts

**DOI:** 10.1289/ehp.9759

**Published:** 2007-04-11

**Authors:** Michelle L. Bell, Keita Ebisu, Kathleen Belanger

**Affiliations:** 1 School of Forestry and Environmental Studies, Yale University, New Haven, Connecticut, USA; 2 Department of Biostatistics and; 3 Department of Epidemiology and Public Health, School of Medicine, Yale University, New Haven, Connecticut, USA

**Keywords:** Air pollution, birth weight, carbon monoxide, nitrogen dioxide, particulate matter, PM_10_, PM_2.5_, pregnancy, sulfur dioxide

## Abstract

**Background:**

Several studies have examined whether air pollution affects birth weight; however results vary and many studies were focused on Southern California or were conducted outside of the United States.

**Objectives:**

We investigated maternal exposure to particulate matter with aerodynamic diameter < 10, < 2.5 μm (PM_10_, PM_2.5_), sulfur dioxide, nitrogen dioxide, and carbon monoxide and birth weight for 358,504 births in Massachusetts and Connecticut from 1999 to 2002.

**Methods:**

Analysis included logistic models for low birth weight (< 2,500 g) and linear models with birth weight as a continuous variable. Exposure was assigned as the average county-level concentration over gestation and each trimester based on mother’s residence. We adjusted for gestational length, prenatal care, type of delivery, child’s sex, birth order, weather, year, and mother’s race, education, marital status, age, and tobacco use.

**Results:**

An interquartile increase in gestational exposure to NO_2_, CO, PM_10_, and PM_2.5_ lowered birth weight by 8.9 g [95% confidence interval (CI), 7.0–10.8], 16.2 g (95% CI, 12.6–19.7), 8.2 g (95% CI, 5.3–11.1), and 14.7 g (95% CI, 12.3–17.1), respectively. Lower birth weight was associated with exposure in the third trimester for PM_10_, the first and third trimesters for CO, the first trimester for NO_2_ and SO_2_, and the second and third trimesters for PM_2.5_. Effect estimates for PM_2.5_ were higher for infants of black mothers than those of white mothers.

**Conclusions:**

Results indicate that exposure to air pollution, even at low levels, may increase risk of low birth weight, particularly for some segments of the population.

Low birth weight is an important predictor of children’s health and is associated with higher risk of infant and childhood mortality (McCormick 1985), coronary heart disease (Vos et al. 2006), and other health problems (Ashdown-Lambert 2005). For example, in a cohort of 10,803 singleton births, Lawlor et al. (2005a) found an inverse relationship between birth weight and coronary heart disease and stroke. Identified risk factors for low birth weight include mother’s age (Khoshnood et al. 2005), prenatal care (Shi et al. 2004), maternal smoking and educational status (Kleinman and Madans 1985), race (Alexander et al. 2003), and socioeconomic factors (Valero de Bernabe et al. 2004). Several studies examined whether maternal exposure to air pollution adversely impacts birth outcomes, such as low birth weight, preterm delivery, intrauterine growth restriction, and postneonatal infant mortality (Glinianaia et al. 2004; Maisonet et al. 2004; Šrám et al. 2005; Woodruff et al. 2006).

Results regarding the relationship between air pollution and birth weight are inconclusive, with some studies identifying associations where others did not, and the suite of adverse pollutants and exposure periods differing by study. For instance, higher levels of carbon monoxide were associated with low birth weight in southern California; six Northeastern U.S. cities; São Paulo, Brazil; Sydney, Australia; and Seoul, South Korea (Gouveia et al. 2004; Ha et al. 2001; Maisonet et al. 2001; Mannes et al. 2005; Ritz et al. 2000; Salam et al. 2005; Wilhelm and Ritz 2003, 2005). However no association was identified in studies based in the Czech Republic, Taiwan, Nevada, and California (Bobak 2000; Chen et al. 2002; Lin et al. 2004; Parker et al. 2005). Particulate matter (PM) with an aerodynamic diameter < 10 μm (PM_10_) was linked with low birth weight in São Paulo, Southern California, Taiwan, the Czech republic, and Seoul (Dejmek et al. 1999; Gouveia et al. 2004; Ha et al. 2001; Wilhelm and Ritz 2005; Yang et al. 2003), with no such evidence provided by other work in Taiwan and six U.S. cities (Lin et al. 2004; Maisonet et al. 2001) and limited evidence in Nova Scotia, Canada (Dugandzic et al. 2006).

Three recent reviews summarized scientific evidence regarding the association between air pollution and birth weight. One review concluded that the effects of air pollution on low birth weight are not fully apparent and that current scientific knowledge is limited (Maisonet et al. 2004). Another determined that PM has either a small effect on fetal growth or no effect, and recommended further research (Glinianaia et al. 2004). The most recent review concluded that existing literature supports a causal link between air pollution and birth weight, although additional research is needed to confirm the effect, investigate the exposure window of importance, and distinguish which pollutants cause harm (Šrám et al. 2005).

The seemingly conflicting evidence may result from inadequate control for confounders, variation in populations and pollution characteristics, or differences in study design such as modeling structure, exposure time frame, and sample size. Residential mobility may differ by study population, resulting in varying levels of exposure misclassification. Effect estimates for PM and mortality and hospital admissions show spatial and temporal heterogeneity, which may be related to dissimilar chemical composition. In particular, the risk of cardiovascular admissions for the elderly from PM with an aerodynamic diameter < 2.5 μm (PM_2.5_) is higher in the eastern United States, including the Northeast region (Dominici et al. 2006), and mortality effects of PM_10_ are strongest in the northeastern United States (Peng et al. 2005). Variation in PM composition may partially explain differing results from studies of PM and low birth weight.

Many study areas for air pollution and birth weight research are outside the United States, such as those areas listed above as well as Lithuania (Maroziene and Grazuleviciene 2002), Zimbabwe (Mishra et al. 2004), Canada (Liu et al. 2003), Croatia (Mohorovic 2004), Poland (Jedrychowski et al. 2004), and China (Wang et al. 1997). Of the U.S.-based studies, most focused on Southern California (Basu et al. 2004; Parker et al. 2005; Ritz and Yu 1999; Salam et al. 2005; Wilhelm and Ritz 2003, 2005). Only one study investigated the northeastern United States, using births from six cities over a 3-year period, and found adverse effects of CO and sulfur dioxide, but not PM_10_ (Maisonet et al. 2001). To the best of our knowledge, no previous study explored the impacts of nitrogen dioxide or fine PM (PM_2.5_) on birth weight in the northeastern United States. In this research we investigated the effects of air pollution on birth weight in Connecticut and Massachusetts over a 4-year period for SO_2_, NO_2_, CO, PM_10_, and PM_2.5_ and explored effects by gestational and trimester exposure and by race.

## Materials and Methods

### Birth data

Birth records data were obtained from the Division of Vital Statistics, Reproductive Statistics Branch of the National Center for Health Statistics (2006) for all registered births in Connecticut and Massachusetts from 1 January 1999 to 31 December 2002 (*n* = 495,260). Each observation contained variables for the residence and birth location by county; prenatal care; mother’s characteristics (age, race, marital status, education, and alcohol and tobacco use during pregnancy); father’s characteristics (age and race); birth order; gestational age in weeks; sex of child; and birth weight.

We excluded births with unspecified county location (0.8%), plural deliveries (4.3%), births with gestational period > 44 weeks or < 32 weeks (2.7%), births with weight < 1,000 g or > 5,500 g (1.2%), and births with impossible gestational age and birth weight combinations (0.5%) (Alexander et al. 1996). Also omitted were births for which the counties of mother’s residence and birth were not identical or in adjacent states (3.9%).

We used gestational period and last menstrual period (LMP), corrected for conception at an average of 2 weeks past LMP, to estimate air pollution exposure for each pregnancy and pollutant for the total pregnancy and each trimester. Various definitions of trimesters have been applied, and some studies have not reported trimester specifications. We used trimester divisions of 1–13 weeks, 14–26 weeks, and 27 weeks to birth; similar definitions have been applied elsewhere (e.g., Parker et al. 2005). Births were omitted if LMP was missing (10.1%) or the date of birth estimated through LMP and gestational period was > 30 days from the midday of the birth month reported on the birth certificate, because this condition implies inaccurate data (10.4%).

### Pollution and weather data

Monitoring data from the U.S. Environmental Protection Agency (U.S. EPA 2006a) was used to estimate daily county-level concentrations for NO_2_, SO_2_, PM_2.5_, PM_10_, and CO. We included air pollution data for 1998 in addition to the years in which eligible births occurred, 1999–2002, to estimate exposure for births in 1999. Each county’s daily average was based on measurements from a single monitor or average of data from multiple monitors within the county. We then used this information to estimate exposure to each pollutant over the gestational period and each trimester using the county of mother’s residence. The frequency of measurement and number of monitors varied by pollutant and county. PM_2.5_ and PM_10_ were measured an average of every 2.7 and 5.5 days, respectively. Other pollutants were measured approximately every day. Some monitors began or ceased operation from 1998–2002, so not all counties had pollutant concentrations for the entire study period.

We excluded births for which air pollutant data were not available for ≥ 75% of the weeks in each trimester for that pollutant (21.5%). This prevents inaccurate exposure estimates due to data gaps or seasonal measurements. For example, exposure for a gestational period of June to February cannot be appropriately based on measurements taken only from July to September. We generated a weighted average of the concentrations on days with data available to account for missing data and changes in frequency measurement. For instance, PM_10_ was measured daily in Hartford County, Connecticut, from January 1998 through May 1999 and measured every 6 days thereafter. A simple average of available data for a gestational period of April to December 1999 would be biased toward the first trimester of exposure. We first generated weekly averages and then overall exposure averages, to avoid this problem.

Temperature has been associated with birth weight for all trimesters and explains much of the seasonal variability (Lawlor et al. 2005b; Murray et al. 2000). Daily county-level temperature and dew point temperature data were obtained from the National Climatic Data Center (2006). Average temperature and dew point temperature exposures were generated based on the same procedure described for air pollution. Apparent temperature, also called the temperature–heat index, was calculated for each trimester to reflect overall temperature discomfort, accounting for temperature and humidity (Kalkstein and Valimont 1986).

After all exclusion criteria, 358,504 births remained in the data set, with most exclusions attributed to lack of sufficient pollution data. Multiple exclusion criteria may apply to a single observation. Characteristics of excluded births are summarized in the Supplemental Material (Table S1; http://www.ehponline.org/docs/2007/9759/suppl.pdf).

Analysis included 15 counties: Fairfield, Hartford, New Haven, New London, and Windham, Connecticut; and Barnstable, Berkshire, Bristol, Essex, Hampden, Middlesex, Norfolk, Plymouth, Suffolk, and Worcester, Massachusetts; with most observations from Massachusetts (71.5%). Air pollutant data were available for 7 counties for CO and for SO_2_, 10 for NO_2_ and PM_10_, and 13 for PM_2.5_.

### Modeling design

We applied a linear model with birth weight as a continuous variable and a logistic model comparing low weight (< 2,500 g) and non-low weight (≥ 2,500 g) births. These methodologies were applied in previous air pollution and birth weight research for the linear model (e.g., Basu et al. 2004; Parker et al. 2005; Woodruff et al. 2003) and logistic version (e.g., Maisonet et al. 2001; Ritz and Yu 1999; Wilhelm and Ritz 2003).

We adjusted for mother’s marital status (married/unmarried), tobacco use during pregnancy (yes/no), alcohol use during pregnancy (yes/no), and education (< 12 years, 12 years, 13–15 years, > 15 years, unknown). To incorporate the nonlinear relationship between mother’s age and birth weight, models contained categorical variables of mother’s age (< 20, 20–24, 25–29, 30–34, 35–39, > 39 years). Mother’s race was indicated as white, black, or other. The “other” category incorporates races for which separate analysis was prohibited by sample size (e.g., Chinese, Filipino, and Vietnamese). Covariates included apparent temperature by trimester, and indicator variables for child’s sex, type of delivery (primary cesarean section, repeat cesarean section, vaginal birth), the time frame in which pre-natal care began (first, second, or third trimester; no care; missing data), first in birth order (yes/no), gestational length (32–34, 35–36, 37–38, 39–40, 41–42, 43–44 weeks), and year of birth.

Covariates were chosen based on previous literature identifying potential risk factors for low birth weight. We first applied linear and logistic models for gestational exposure including all potential covariates listed above and excluding air pollution variables to explore whether expected associations were observed (e.g., maternal smoking associated with lower birth weight). Covariates exhibiting a statistically significant relationship with birth weight were incorporated into models investigating air pollution exposure.

We also applied the linear model using exposures for each trimester. Because trimesters’ exposure can be correlated, we performed sensitivity analysis with a model that used trimester exposures as: *a*) *P_a,j_^i^* = exposure to pollutant *j* over trimester *a* for birth *i; b*) residuals of *E*[*P^i^_b,j_* ] = β _1_ + β _2_*P_a,j_^ii^*, representing exposure to pollutant *j* over trimester *b* for birth *i*, adjusted for exposure over trimester *a*; and *c*) residuals of the model *E* [*P_c,j_^i^*] = β_3_+ β_4_*P^i^_a,j_* + β_5_*P^i^_b,j_* , representing exposure to pollutant *j* over the trimester *c* for birth *i*, adjusted for exposure over trimesters *a* and *b*. The β values are regression coefficients, in which β _1_ and β _3_ represent intercepts, β _2_ represents the association between exposure in trimesters *a* and *b* (i.e., change in exposure in trimester *b* associated with a unit increase in exposure in trimester *a*), β _4_ represents the association between exposure in trimesters *a* and *c* adjusted for exposure in trimester *b*, and β _5_ represents the association between exposure in trimesters *b* and *c* adjusted for exposure in trimester *a.* We repeated this analysis using each trimester as the initial reference trimester (i.e., trimester *a*). This approach avoids covariance among variables representing trimester exposures.

We conducted sensitivity analysis for the linear and logistic gestational exposure models restricting the data set to first births only. Two pollutant linear models were implemented for gestational exposures to explore potential confounding by co-pollutants. To investigate whether air pollution’s effects on birth weight differ by race, we applied an interaction model of race and gestational exposure for each air pollutant.

## Results

Pollutant exposure was estimated for 358,504 births, although not all births had data for all pollutants. Low birth weight (< 2,500 g) comprised 4.0% of total births, 3.6% of male births, and 4.5% of female births. Table 1 provides descriptive statistics of the study population, weather, and pollutant exposures. Birth weights followed a seasonal pattern with highest weights in spring (mean, 3431.0 g) and lowest in winter (3409.4 g). Figure S1 in the Supplemental Material (http://www.ehponline.org/docs/2007/9759/suppl.pdf) shows monthly patterns of pollutant levels and the percentage of births that were low birth weight (< 2,500 g).

Parents were predominantly married and white, with mean mother’s and father’s ages of 29.5 and 32.3 years, respectively. Father’s race and age were not included in the analysis, but were strongly related to mother’s race and age. Correlations between father’s and mother’s races were 0.78 to 0.82 for the three race categories considered (black, white, and other), and 86.3% of mother–father pairs had the same race classification. Father’s and mother’s ages were correlated at 0.75. Exposure estimates for some pollutants covaried. Gestational exposure to PM_2.5_ had 0.77 and 0.64 correlations with PM_10_ and NO_2_, respectively. The correlation between exposures to NO_2_ and PM_10_ was 0.55.

Table 2 shows results for selected variables for the linear model including all variables except air pollution. Lower birth weights were associated with female infants, shorter gestational periods, maternal tobacco use, less maternal education, prenatal care beginning later in pregnancy, first in the birth order, and unmarried, older, or younger mothers. The logistic model provided similar results. All covariates other than alcohol exhibited statistically significant associations. Models examining the effects of air pollution were adjusted for apparent temperature and indicators for year, as well as all covariates listed in Table 2, except for maternal alcohol use.

Table 3 shows single-pollutant linear and logistic model results for the associations between birth weight and gestational exposure to air pollutants. NO_2_ and PM_2.5_ were linked to lower birth weight in the linear model and increased odds of low birth weight in the logistic model. Associations were also observed for PM_10_ and CO in the linear model. No relationship with birth weight was identified for gestational SO_2_ exposure.

**Figure 1:**
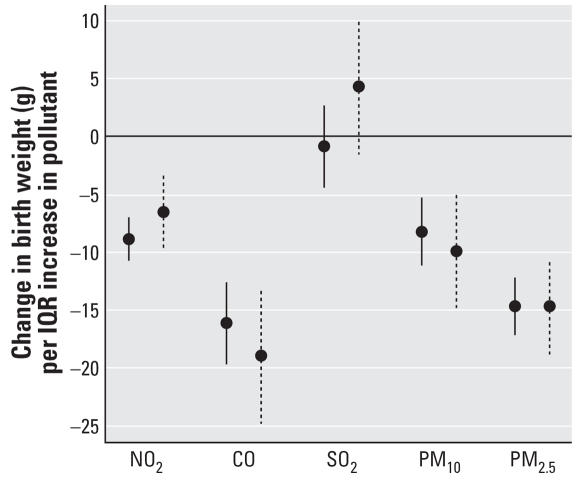
Change in birth weight per IQR increase in gestational exposure, using the linear model with all births (solid line, *n* = 358,504) and first births only (dashed line, *n* = 129,282). The point reflects the central estimate; the vertical line represents the 95% confidence interval.

Sensitivity analysis using first births only (*n* = 129,282) for the linear model provided similar results to those based on all data (*n* = 358,504) (Figure 1). For the logistic model, analysis of first births only did not show statistically significant associations; the increase in risk of low birth weight per interquartile range (IQR) increase in pollutant was 2.01% for NO_2_ and 3.44% for PM_2.5_, compared with 2.65% and 5.39% using the full data set.

**Figure 2:**
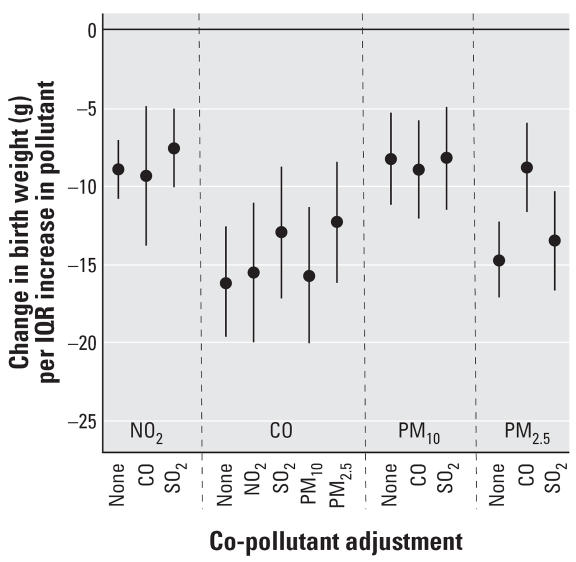
Change in birth weight per IQR increase in gestational exposure to pollutant, for single and two-pollutant linear models. The point reflects the central estimate; the vertical line represents the 95% confidence interval.

Two-pollutant linear models were applied for gestational exposure for pollutants exhibiting significant associations in Table 3, excluding pairs of pollutants that covaried. Figure 2 compares results with and without co-pollutant adjustment. Results for all pollutants considered in the multivariate analysis were robust to co-pollutant adjustment, remaining statistically significant in all cases.

Exposures among trimesters were often related. Correlations among trimester exposures were 0.48, 0.70, and 0.72 for CO; 0.51, 0.76, and 0.77 for NO_2_; 0.63, 0.66, and 0.67 for PM_10_; 0.10, 0.19, and 0.60 for PM_2.5_; and –0.60, 0.08, and 0.20 for SO_2_. We performed analysis including all trimesters of exposure in the linear model, and analysis of adjusted trimester exposures as described in “Materials and Methods” (correlation among adjusted trimester exposures for the same pollutant < 0.005). Table 4 shows the relationship between birth weight and trimester exposure for results that were consistent across all trimester models. The most important trimester of exposure was the third for PM_10_, and the first and third for CO, the first for NO_2_ and SO_2_, and the second and third for PM_2.5_.

To investigate whether effect estimates for pollution and birth weight differ by race, we applied linear models including an interaction term for race and gestational exposure (Table 5). Lower birth weight was associated with PM_2.5_ for infants of either black or white mothers. However, the effects were higher for infants of black mothers (i.e., the regression coefficients were statistically different). No statistical difference was observed between the effect estimates for infants of black and white mothers for the other pollutants.

## Discussion

Exposure to air pollution has been linked to low birth weight in various studies, including exposure at each trimester. Table 6 compares our results with those of previous U.S.-based studies. For comparability, results from other studies were converted to a common metric based on the IQR for gestational exposure for our data set (see Table 1).

A previous study of six northeastern U.S. cities found a stronger relationship between CO exposure and low birth weight for African-American infants than for whites (Maisonet et al. 2001). We identified no difference between the effects of CO by race, although effect estimates were higher for infants of black mothers for PM_2.5_. This indicates that some populations may face disproportionate health burdens of air pollution. This may result from differences in baseline health status, health care service, or occupational exposure. Effect modification by race may also be due to differential exposure such as higher traffic pollution relating to residential proximity to highways, because effects were statistically different by race for PM_2.5_, a traffic-related pollutant. Factors pertaining to occupation and health care status could be explored in future studies, such as a cohort study with detailed information on each observation.

Relationships between gestational exposure and birth weight were robust to inclusion of another pollutant (Figure 2). However, only pollutant pairs that were uncorrelated were included in two pollutant models. Exposure to PM_10_, PM_2.5_, and NO_2_ were highly correlated in this data set, resulting from common sources. Thus this research cannot distinguish between detrimental effects of various pollutants (e.g., effects of NO_2_ versus PM_2.5_); pollutants identified in this work could be functioning as a proxy for other forms of pollution sharing similar sources. Additional research is needed to better isolate the key pollutants of harm. In particular, differences in chemical composition may help explain variation in results across study areas. For instance, PM_2.5_ in the eastern United States has a higher contribution of sulfate whereas PM_2.5_ in the western United States has a larger nitrate component (Bell et al. 2007).

Several studies examined how variation in air pollution levels can affect epidemiologic research. In Los Angeles, PM_2.5_ exhibited within-city gradients, which produced larger effect estimates for mortality than models comparing PM_2.5_ across communities (Jerrett et al. 2005). Residential proximity to traffic was associated with low birth weight and preterm delivery in Los Angeles (Wilhelm and Ritz 2003, 2005). A California study found that various air pollution metrics (county-wide average, nearest monitor, distance-weighted average of monitors < 5 miles of mother’s residence) produced different effect estimates for the relationship between air pollution and birth weight (Basu et al. 2004). These studies suggest that heterogeneity in air pollution concentrations can exist at sub-county scales and that this variation can affect health effects estimates, which may affect our study based on countywide estimates. Additionally, we used ambient monitoring as a surrogate for personal exposure, which does not address indoor/outdoor activity patterns and occupational exposure. Nondifferential misclassification, such as from subcounty heterogeneity in pollution concentrations, would drive effect estimates toward the null, so any effect estimates would more likely underestimate true effects.

Our adjustment for mother’s educational status may not fully address potential confounding by socioeconomic status. Actual socioeconomic position is related not only to education but to numerous other factors such as income, social class, and historical socioeconomic status (Krieger et al. 1997). Similarly, our binary measure of tobacco use during pregnancy may not have fully captured its effects, and use of alcohol and tobacco was based on self-reporting. The misclassification of a covariate can bias results (Marshall and Hastrup 1996; Marshall et al. 1999). The use of more detailed information is preferable, such as number of cigarettes smoked during pregnancy rather than a yes/no category (Greenland 2001). Associations may be biased if covariate definitions are based on occurrence anytime during pregnancy (e.g., smoking as a yes/no variable) and the actual association between the covariate and birth weight occurs during a vulnerable period of pregnancy, such as the third trimester (Hertz-Picciotto et al. 1996).

We estimated the time frame for exposure on the basis of LMP as documented on birth certificates; however, LMP is self-reported. As an example, the 10th, 15th, and 20th days of the month should each account for about 3.3% of total LMP days, but were reported as the approximate LMP date more often at 4.4%, 5.6%, and 4.8%, respectively. Exposure misclassification as a function of date of birth and gestational period is less likely to affect exposure estimates for the entire pregnancy than for individual trimesters, based on the longer time frame. For example, if the actual LMP is a week earlier than reported, exposure will be misclassified for about 2.6% of the gestational period and about 7.7% of a trimester, for a typical length pregnancy.

The exposure estimates assume that the county of mother’s residence at the time of birth is constant throughout gestation. A limited number of studies have investigated residential mobility during pregnancy. Although they are based on small sample sizes (295–398 births), results indicated that 12–24.8% of mothers moved during pregnancy, but that only 4–7.7% moved to a different county (Fell et al. 2004; Khoury et al. 1988; Shaw and Malcoe 1992). One study concluded that exposure based on residence at delivery is a reasonable estimate of exposure during pregnancy for exposures at county-level resolution (Shaw and Malcoe 1992). Based on results of these residential mobility studies, misclassification of exposure introduced by the mother moving during pregnancy is unlikely to greatly influence our results. Such misclassification is more likely to affect results for the first and second trimesters than the third because they are further from the date at which residence was obtained (i.e., date of delivery). Future research incorporating detailed maternal history including residence throughout the pregnancy would improve pollution exposure estimates as well as advance adjustment by potential confounders such as socioeconomic factors.

Factors associated with higher mobility during pregnancy include lower education (Fell et al. 2004; Shaw and Malcoe 1992); mother ≤ 25 years (Fell et al. 2004), ≤ 20 years (Shaw and Malcoe 1992), 20–24 years (Khoury et al. 1988), or ≥ 35 years of age (Fell et al. 2004); low family income (Fell et al. 2004); unmarried mother (Fell et al. 2004); maternal tobacco use (Fell et al. 2004); and white mother (Khoury et al. 1988). All of the above factors linked with increased mobility, other than white mother, are also associated with low birth weight, leading to the potential for more exposure misclassification for mothers of lower weight infants.

The physiological mechanism by which air pollution could affect birth weight is not fully understood, although several theories have been proposed. The pathways could be similar to those of maternal smoking, which can increase risk of preterm delivery through premature rupture of membranes and placental abruption and lower birth weight. Air pollution could affect fetal health either through direct effects on the fetus by exposure through the placenta or from effects on the mother’s health (Glinianaia et al. 2004), and multiple mechanisms may occur simultaneously. Maternal pulmonary function has been linked to altered placental vascular function and growth retardation in asthmatic pregnancies (Bracken et al. 2003; Clifton et al. 2001; Schatz et al. 1990). Other theories include *a*) altered cardiac function from changes in heart rate variability; *b*) inhalation by the mother of polycyclic aromatic hydrocarbons (PAHs) that then relate to placental exposure, potentially disrupting endocrine and nervous systems; *c*) changes in blood viscosity due to alveolar inflammation from PM, which in turn affects placental function; and *d*) binding of CO to hemoglobin binding sites, preventing the binding of oxygen and subsequent function (Glinianaia et al. 2004; Maisonet et al. 2004; Šrám et al. 2005).

In summary, this research indicates that maternal exposure to air pollution may adversely affect risk of low birth weight, even in areas without high pollution levels. Average concentrations for all pollutants in the study were below the U.S. EPA health-based National Ambient Air Quality Standards (NAAQS). Further, all counties in the study are currently in compliance with the NAAQS for NO_2_, CO, SO_2_, and PM_10_. Only two counties, New Haven and Fairfield, Connecticut, are in noncompliance with the NAAQS for PM_2.5_ (U.S. EPA 2006b).

## Figures and Tables

**Table 1:** Descriptive statistics of the study population and exposures (*n* = 358,504).

Variables relating to birth and mother	Value
Birth weight (g)	3422.7 ± 514.9
Low birth weight [< 2,500 g (%)]	4.01
Child’s sex (%)	
Male	51.1
Female	48.9
Type of birth (%)	
Primary cesarean section	13.9
Repeat cesarean section	8.4
Vaginal birth	77.4
Unknown	0.3
First child (%)	
Yes	36.1
No	63.9
Month prenatal care began (%)	
First 3 months of pregnancy	89.4
4th–6th month of pregnancy	8.4
7th month of pregnancy or later	1.8
No care	0.2
Unknown	0.3
Gestational length (weeks)	39.1 ± 1.73
32–34 (%)	1.7
35–36 (%)	5.0
37–38 (%)	23.7
39–40 (%)	51.1
41–42 (%)	16.6
43–44 (%)	1.8
Alcohol use by mother (%)	
Yes	1.6
No	97.5
Unknown	0.9
Tobacco use by mother (%)	
Yes	8.7
No	90.5
Unknown	0.9
Mother’s education (%)	
< 12 years	12.2
12 years	24.8
13–15 years	22.4
> 15 years	39.9
Unknown	0.7
Mother’s race (%)	
White	83.4
Black	10.7
Other	6.0
Mother’s marital status (%)	
Married	72.7
Unmarried	27.3
Mother’s age (years)	29.5 ± 6.0
< 20 (%)	6.7
20–24 (%)	15.3
25–29 (%)	24.3
30–34 (%)	32.7
35–39 (%)	17.5
> 39 (%)	3.5
Season and weather	
Season of birth (%)	
Winter	23.1
Spring	24.7
Summer	26.6
Fall	25.6
Temperature (°F)	50.5 ± 5.6
Dew point temperature (°F)	40.0 ± 5.8
Gestational pollution exposures (mean ± SD)	
NO_2_ (IQR 4.8 ppb)	17.4 ± 5.0 ppb
CO (IQR 303 ppb)	656 ± 180 ppb
SO_2_ (IQR 1.6 ppb)	4.7 ± 1.2 ppb
PM_10_ (IQR 7.4 μg/m^3^)	22.3 ± 5.3 μg/m^3^
PM_2.5_ (IQR 2.2 μg/m^3^)	11.9 ± 1.6 μg/m^3^

Values are percentages or mean ± SD.

**Table 2:** Difference in birth weight associated with selected nonpollution variables (95% confidence interval).

Variable	Difference in birth weight (g)
Child’s sex	
Female (reference)	
Male	133.1 (130.1 to 136.1)
Mother’s education (years)	
12 (reference)	
< 12	–32.7 (–38.3 to –27.1)
13–15	22.9 (18.4 to 27.3)
> 15	34.1 (29.7 to 38.4)
Unknown	–36.1 (–56.1 to –16.1)
Tobacco use by mother	
No (reference)	
Yes	–176.5 (–182.2 to –170.9)
Alcohol use by mother	
No (reference)	
Yes	–9.4 (–21.5 to 2.6)
Mother’s marital status	
Married (reference)	
Unmarried	–46.5 (–50.9 to –42.1)
Mother’s race	
White (reference)	
Black	–97.8 (–102.9 to –92.7)
Other	–176.3 (–182.7 to –169.8)
Month prenatal care began	
Month 1–3 (reference)	
Month 4–6	–29.2 (–34.8 to –23.6)
Month ≥ 7	–50.2 (–61.7 to –38.7)
No care	–102.8 (–140.1 to –65.4)
Mother’s age (years)	
30–34 (reference)	
< 20	–41.8 (–49.7 to –34.0)
20–24	–39.4 (–44.8 to –34.1)
25–29	–16.4 (–20.6 to –12.3)
35–39	2.9 (–1.6 to 7.4)
> 39	–19.8 (–28.3 to –11.3)
Birth order	
Firstborn (reference)	
Not firstborn	101.9 (98.4 to 105.3)
Gestational length (weeks)	
39–40 (reference)	
32–34	–1050.2 (–1061.8 to –1038.5)
35–36	–585.2 (–592.3 to –578.2)
37–38	–227.7 (–231.5 to –224.0)
41–42	122.3 (118.0 to 126.6)
43–44	62.4 (51.0 to 73.8)

*p* < 0.01 for all associations except alcohol use.

**Table 3:** Change in birth weight per IQR increase in pollution for the gestational period (95% confidence interval).

Pollutant	Difference in birth weight (g)	Odds ratio for low birth weight (< 2,500 g)
NO_2_	–8.9 (–10.8 to –7.0)*	1.027 (1.002 to 1.051)**
CO	–16.2 (–19.7 to –12.6)*	1.028 (0.983 to 1.074)
SO_2_	–0.9 (–4.4 to 2.6)	1.003 (0.961 to 1.046)
PM_10_	–8.2 (–11.1 to –5.3)*	1.027 (0.991 to 1.064)
PM_2.5_	–14.7 (–17.1 to –12.3)*	1.054 (1.022 to 1.087)**

* *p* < 0.001;

** *p* < 0.05.

**Table 4:** Change in birth weight per IQR increase in pollutant for trimester exposure.

Pollutant	Trimester	Range among the trimester models for change in birth weight (g) per IQR increase in pollutant
NO_2_	1st	–9.6 to –8.3
CO	1st	–11.3 to –9.9
	3rd	–14.0 to –16.3
SO_2_	1st	–3.7 to –3.3
PM_10_	3rd	–6.6 to –4.7
PM_2.5_	2nd	–7.2 to –5.4
	3rd	–9.0 to –7.0

For comparability, the IQR for gestational exposure was used.

**Table 5:** Change in birth weight per IQR increase in pollutant for the gestational period, by race (95% confidence interval).

	Difference in birth weight (g) per IQR of pollutant	
Pollutant	Black mother	White mother
NO_2_	–12.7 (–18.0 to –7.5)	–8.3 (–10.4 to –6.3)
CO	–10.9 (–20.2 to –1.6)	–17.5 (–21.3 to –13.7)
SO_2_	1.2 (–6.5 to 8.8)	–1.4 (–5.1 to 2.3)
PM_10_	–7.9 (–16.0 to 0.2)	–9.0 (–12.2 to –5.9)
PM_2.5_*	–22.6 (–29.3 to –15.9)	–14.7 (–17.3 to –12.0)

* *p* < 0.05 for comparison of results from infants with black mothers to infants with white mothers.

**Table 6:** Comparison of results from U.S.-based studies.

Study location (time period)	No. of births	Results	Study (confounders considered)
Connecticut and Massachusetts (1999–2002)	358,504	NO_2_: Effect for first trimester, gestational exposure CO: Effect for first and third trimesters, gestational exposure SO_2_: Effect for first trimester PM_2.5_: Effect for second and third trimesters, gestational exposure PM_10_: Effect for third trimester and gestational exposure (see Tables 3 and 4)	This study (1–7,9,11,13,17,19)
Georgia (April 1986–March 1988)	325	PM_10_: 94% (–2 to 283%) increased odds of very low birth weight (< 1,500 g) for exposure > 15.07 μg/m^3^ compared with < 1.48 μg/m^3^	Rogers and Dunlop (2006) (1,3,4,6–9,11–16,18,23,24)
California (2000)	18,247	PM_2.5_: 6.4 g (3.6 to 9.3 g) decrease per 2.2 μg/m^3^. Did not find a particular trimester to be most important CO: No effect	Parker et al. (2005) (1,5–7,9,18)
California (1975–1987)	3,901	CO: 4.7 g (0.24 to 9.2 g) decrease per 303 ppb in first trimester. No effect for gestational exposure PM_10_: 8.0 g (0.41, 15.6 g) decrease per 7.4 μg/m^3^ in third trimester. No effect for gestational exposure	Salam et al. (2005) (2,3,5–7,9,11,18–21,25)
Los Angeles, California (1994–2000)	Up to ~ 115,000*	CO: 3.5% (1.5 to 5.4%) increased odds of low birth weight per 303 ppb in third trimester PM_10_: 2.2% (–2.2 to 6.6%) increased odds of low birth weight per 7.4 μg/m^3^ in third trimester. For mothers ≤ 1 mile of monitoring station effect was 15.6% (3.6 to 10.0%)	Wilhelm and Ritz (2005) (1–4,6,7,9,18,20,22)
California (2000)	16,693	PM_2.5_: 8.9 g (3.0 to 14.8 g) decrease per 2.2 μg/m^3^	Basu et al. (2004) (1,5–7,9]
Los Angeles, California (1994–1996)	~ 48,000*	Lower birth weight associated with distance-weighted traffic density	Wilhelm and Ritz (2003) (1–4,6,7,9,17,18,20,22)
United States (1998–1999)	7,355,696	No effect for small for gestational age based on air pollution index	Woodruff et al. (2003) (1,5–7,9,25)
Northern Nevada (1991–1999)	39,338	PM_10_: 8.1 g (1.7 to 14.7 g) decrease per 7.4 μg/m^3^ in third trimester. No effect in logistic model CO: No effect	Chen et al. (2002) (3–7,9–13,15,17,18,25,27)
Six U.S. Northeastern cities (1994–1996)	101,153	CO: 8.6% (1.8 to 15.7%) increased odds of low birth weight per 303 ppb in third trimester PM_10_: No effect SO_2_: 0.0002% (–0.001 to 0.002%) increased odds of low birth weight per 1.6 ppb in second trimester. Statistically significant effects identified when comparing strata of SO_2_ levels	Maisonet et al. (2001) (1–7,9,11,13,15,18,28)
Georgia (April 1986–March 1988)	345	TSP and SO_2_: 188% (16 to 613%) increased odds of very low birth weight for exposure > 56.75 μg/m^3^ compared with < 9.94 μg/m^3^	Rogers et al. (2000) (1–4,6–9,11–16,23–26)
Los Angeles, California (1989–1993)	125,573	CO: 22% (3 to 44%) increased odds of low birth weight for exposure ≥ 5.5 ppm compared with < 2.2 ppm for third trimester	Ritz and Yu (1999) (1–4,6,7,9,20,29,30)
Denver, Colorado (1975–1983)	2,870	CO: No effect for third trimester	Alderman et al. (1987) (1–7,9)

No effect indicates the lack of a statistically significant effect. Confounders considered may be addressed by inclusion of a variable in models, restriction, or stratification. Gestational length is listed if it was included in modeling; gestational age was also addressed in some studies by including only term births. Confounders: 1 = parity, 2 = gestational length, 3 = sex, 4 = prenatal care, 5 = maternal marital status, 6 = maternal age, 7 = maternal socioeconomic status (education, financial stress, or income), 8 = paternal education, 9 = maternal race, 10 = paternal race, 11 = maternal tobacco use, 12 = maternal drug use, 13 = maternal alcohol use, 14 = maternal exposure to passive smoke, 15 = maternal weight gain, 16 = maternal pre-pregnancy weight, 17 = year, 18 = season or month of birth, 19 = weather, 20 = time since last live birth, 21 = gestational diabetes, 22 = previous low birth weight delivery, 23 = maternal working status, 24 = toxemia, 25 = community, 26 = urban or rural area, 27 = risk factors (e.g., anemia, uterine bleeding), 28 = previous terminations, 29 = restricted to no maternal hypertension, uterine bleeding, or diabetes, 30 = commute time, percent walking to work, and percent of women working with children considered as ecologic variables.

* Sample size varies by analysis.
